# The impact of AMIGO2 on prognosis and hepatic metastasis in gastric cancer patients

**DOI:** 10.1186/s12885-022-09339-0

**Published:** 2022-03-16

**Authors:** Keisuke Goto, Masaki Morimoto, Mitsuhiko Osaki, Akimitsu Tanio, Runa Izutsu, Yoshiyuki Fujiwara, Futoshi Okada

**Affiliations:** 1 Division of Experimental Pathology, Faculty of Medicine, Tottori University, 86 Nishicho, Yonago, 683-8503 Japan; 2 Department of Surgery, Division of Gastrointestinal and Pediatric Surgery, Faculty of Medicine, Tottori University, 36-1 Nishi-cho, Yonago, 683-8504 Japan; 3 Chromosome Engineering Research Centre, Tottori University, 86 Nishicho, Yonago, 683-8503 Japan

**Keywords:** AMIGO2, AMIGO, Gastric cancer, Hepatic metastasis

## Abstract

**Background:**

Gastric cancer (GC) is one of the most common malignancies, and the liver is the most common site of hematogenous metastasis of GC. AMIGO2 is a type I transmembrane protein that has been implicated in tumour cell adhesion in adenocarcinomas; however, its importance in GC remains undetermined.

**Methods:**

We analyzed AMIGO2 expression by immunohistochemistry using the specific monoclonal antibody for human AMIGO2 in 128 patients who underwent GC surgery to evaluate its relationship between various metastatic and clinical outcomes in GC.

**Results:**

Immunohistochemistry revealed that AMIGO2 expression was an independent prognostic factor for overall survival, disease-specific survival, and liver metastasis in GC patients.

**Conclusions:**

This study showed that AMIGO2 is induced in GC tissues and can mediate hepatic metastasis. Determining AMIGO2 expression in GC will help predict patient prognosis and the incidence of liver metastasis.

**Supplementary Information:**

The online version contains supplementary material available at 10.1186/s12885-022-09339-0.

## Background

As of 2020, gastric cancer (GC) is the fifth most common cancer worldwide in terms of incidence and the fourth leading cause of cancer-related mortality [1]. Thus, GC remains an important malignancy that has a poor prognosis, with an estimated annual incidence of over 1 million new cases and 769,000 deaths [1].

The liver is the most common site of hematogenous metastasis of GC, occurring in 3.5% to 11.1% of GC patients [2–6]. Liver resection for metastatic liver tumours in patients with colorectal and neuroendocrine cancer can be a useful option because of their relatively good prognoses after resection; however, for GC and esophagogastric junction cancer, the 5-year survival rates after liver resection are 27% and 12%, respectively, which are inferior to those of breast, ovarian, and testicular cancers. Furthermore, the diagnostic and therapeutic strategies for liver metastases of all these malignancies remain insufficient [7].

In GC patients, non-curative treatments including systemic chemotherapy and palliative therapy are the standard treatment for liver metastases, and hepatic resection with an R0 margin for a single small nodule can be acceptable to improve the prognosis of synchronous or metachronous liver metastases [8–10]. Additionally, it is difficult to accurately diagnose liver micrometastases preoperatively, and early postoperative recurrence would suggest that subclinical metastases had occurred at the time of surgery [11, 12]. Therefore, to improve the prognosis of GC patients, it is important to elucidate the detailed mechanisms by which GC cells metastasize to the liver and to develop markers that can predict liver metastasis.

The human *AMIGO2* gene was originally identified as a novel transmembrane protein involved in neuronal processes [13]. The AMIGO family consists of AMIGO1/2/3, all of which are type I transmembrane proteins with six leucine-rich repeats and one immunoglobulin-like domain in the extracellular region. No specific ligand of them has been identified. Although the proteins of the AMIGO family share many structural similarities, their expression patterns and biological activities are dissimilar.

In human adenocarcinomas, AMIGO2 has been implicated in tumour cell adhesion [14, 15]. In ovarian cancer, cancer cells with high AMIGO2 expression show a significantly increased growth rate in the abdominal cavity [16]. In GC, patients with high *AMIGO2* mRNA expression have a significantly worse prognosis [17].

However, few studies have investigated AMIGO2 expression using immunohistochemistry in cancer tissues or whether AMIGO2 is related to GC prognosis or metastasis. The intracellular localization of AMIGO2 has not been clarified yet. In this study, we performed immunostaining of primary gastric tumour tissues with the specific monoclonal antibody (rTNK1A0012a) for human AMIGO2 [18] to investigate the relationship between AMIGO2 expression and various metastatic and clinical outcomes.

## Methods

### Patient samples

Immunohistochemical analysis was performed using paraffin-embedded GC samples from 128 patients with GC who underwent gastrectomy at our institution between January 2010 and December 2013. Clinicopathological findings were determined by the Japanese Classification of GC [19]. The Tottori University Ethical Board approved the protocol (approval number: 17A142).

### Immunohistochemistry

Serial sections were cut from formalin-fixed paraffin-embedded tissue samples at 4 µm, and then the sections were deparaffinized in xylene and rehydrated through a graded alcohol series. For antigen retrieval, the sections were incubated in 10 mM citrate buffer (pH 6.0) at 121 °C for 20 min in an autoclave. Sections were then incubated in 0.1% hydrogen peroxidase for 15 min to block endogenous peroxidases and in 10% goat normal serum (424,041; Nichirei Bioscience, Tokyo, Japan) for 15 min to prevent non-specific antigen binding. Since the commercially available antibody sc-373699 was found to be cross-reactive with all three molecules AMIGO1, 2 and 3 [18], in this study, we evaluated the protein expression of AMIGO2 in tumor cells using a newly developed antibody for human AMIGO2 (rTNK1A0012a), with a specificity to only AMIGO2 [18]. Slides were subsequently incubated with the antibody for human AMIGO2; rTNK1A0012a [18], overnight at 4 C, and then with preabsorbed horseradish peroxidase-conjugated goat anti-rat IgG (ab98425; Abcam, Cambridge, UK) for 20 min. Staining was visualized with diaminobenzidine (SK-4105; Vector Laboratories, Burlingame, CA, USA), and then sections were counterstained with haematoxylin. AMIGO2 expression in GC cells was evaluated in a blinded manner. Briefly, five random fields-of-view (400 × magnification) were examined. Tumours with > 70% positive cells were classified as high expression and those with ≤ 70% positive cells were classified as low expression. Two investigators (G.K. and M.M.) evaluated the immune-labelling; agreement was obtained in each case.

### Statistical analysis

Differences between categorical variables were determined using the chi-squared test and Fisher's exact test. The Kaplan–Meier method was used to generate survival curves, and differences in survival curves were compared using the log-rank test. Cox proportional hazards models were used for multivariate analyses. To identify the risk factors of liver metastasis and peritoneal dissemination, multivariate analyses were performed by logistic regression analysis. *p* < 0.05 was considered significant. SPSS statistics version 25.0 software (IBM Corp., Armonk, NY, USA) and EZR software ver1.55 (Saitama Medical Center, Jichi Medical University) were used for all statistical analyses.

## Results

### AMIGO2 expression was associated with poor prognosis in GC patients

**Fig. 1:**
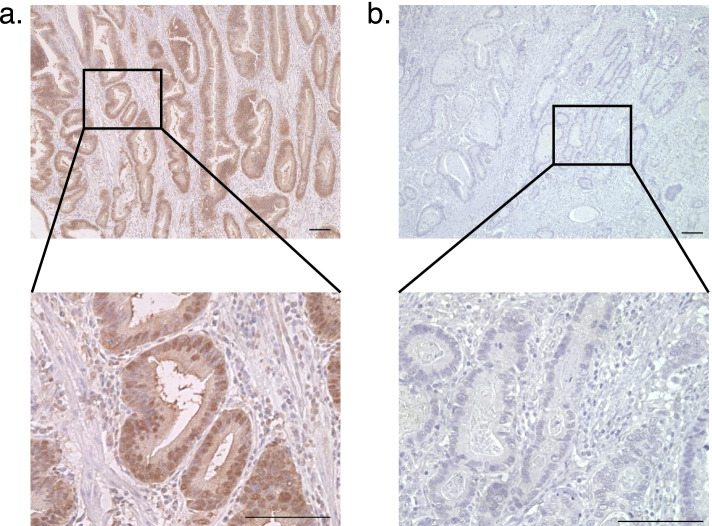
Representative images of the differential staining intensities of AMIGO2 staining in gastric cancer cells. AMIGO2 staining was predominantly cytoplasmic and nuclear in cancer cells. **a** The high (> 70% staining intensity) and (**b**) low (≤ 70% staining intensity) AMIGO2 expression groups. Scale bar: 100 μm

To assess AMIGO2 protein expression in GC tissues and to determine if there was a correlation with prognosis in GC patients, 128 GC specimens were evaluated by immunohistochemistry. Most GC cancer cells were moderately to strongly stained with AMIGO2. Positive staining was predominantly observed in the cytoplasm and nuclei of cancer cells (Fig. 1a, 1b). Cancer cells that were strongly stained in the cytoplasm and plasma membrane were considered to have positive AMIGO2 expression. The cut-off point for high expression was set at 70% staining frequency of cancer cells, and cases were divided into two groups: high expression (> 70%, Fig. 1a) and low expression (≤ 70%, Fig. 1b). On the basis of these criteria, high AMIGO2 expression was found in 22.7% (29/128) of samples.

Table 1 shows the relationship between AMIGO2 expression and the clinicopathological features of GC patients. AMIGO2 expression was significantly higher in male patients (*p* = 0.047) and in those with vascular invasion (*p* = 0.013), whereas other factors including conventional serum tumour markers did not show a significant relationship with AMIGO2 expression (Table 1).

**Table 1:** Association between AMIGO2 expression and clinicopathological factors. The chi-square test and Fisher's exact test were used for the statistical analyses

parameter		AMIGO2 expression		*P* value
		negative	positive	
		*n* = 99	*n* = 29	
Age(years)	< 70	44	14	0.715
	≥ 70	55	15	
Sex	male	71	26	**0.047**^a^
	female	28	3	
BMI	< 25	82	26	0.283
	≥ 25	17	3	
Neoadjuvant chemotherapy	yes	9	2	0.526
	no	90	27	
Adjubant chemotherapy	yes	30	11	0.439
	no	69	18	
Stage (UICC 7th)	I	48	14	0.086
	II	28	3	
	III	20	10	
	IV	3	2	
Histlogical type	poor/sig	54	10	0.057
	well/mod/other	45	19	
Vascular invasion	negative	42	5	**0.013** ^a^
	positive	57	24	
Lymphatic invasion	negative	34	7	0.300
	positive	65	22	
pT	I/II	56	16	0.894
	III/IV	43	13	
pN	0	58	15	0.512
	1	41	14	
CEA (ng/mL)	< 5	78	20	0.272
	≥ 5	21	9	
CA19-9 (U/mL)	< 35	87	26	0.545
	≥ 35	12	3	
Liver metastasis	presence	7	8	**0.006** ^a^
	absence	92	21	
Peritoneal dissemination	presence	12	8	**0.047** ^a^
	absence	87	21	

*BMI* body mass index, *sig/poor/mod/well* signet-ring cell/poorly/moderately/well differentiated, *pT/N* pathological T/N stage, *CEA* carcinoembryonic antigen, *CA19-9* carbohydrate antigen19-9, ^a^statistically significant, the chi-square test and Fisher's exact test were used for the statistical analyses

**Fig. 2:**
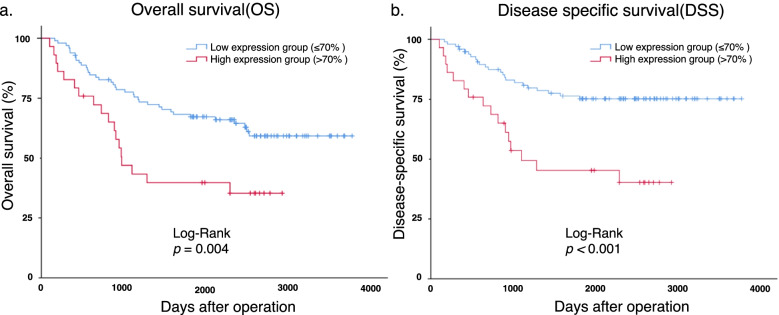
AMIGO2 expression is negatively correlated with prognosis of patients with gastric cancer. **a** Kaplan–Meier analysis of overall survival in gastric cancer patients with high (> 70% staining intensity) vs. low (≤ 70%) AMIGO2 expression. Differences between the survival curves were examined using the log‑rank test. **b** Kaplan–Meier analysis of disease-specific survival in gastric cancer patients with high (> 70% staining intensity) vs. low (≤ 70%) AMIGO2 expression. Differences between the survival curves were examined using the log‑rank test

To clarify the relationship between AMIGO2 expression and the prognosis of GC patients, Kaplan–Meier curves were generated and the log-rank test was performed. Patients with high AMIGO2 expression had worse overall survival (OS; *p* = 0.004, Fig. 2a) and disease-specific survival (DSS; *p* < 0.001, Fig. 2b) compared with patients with low AMIGO2 expression. Additionally, administration of neoadjuvant/adjuvant chemotherapy, stage II/III/IV disease, positivity for vascular/lymphatic invasion, and pathological T/N stages (pT/N) were risk factors for poorer OS and DSS after surgery in this cohort (Table 2). The prognostic curve for each stage was presented in supplementary Figure 1.

**Table 2:** Survival analyses of prognostic factors

		No. patients	OS			DSS		
			MST (days)	95% CI	*P* value	MST (days)	95% CI	*P* value
Age (years)	< 70	58	NA	NA	0.164	NA	NA	0.272
	≥ 70	70	2497	NA		NA	NA	
Sex	male	97	NA	NA	0.315	NA	NA	0.487
	female	31	2473	981–3965		NA	NA	
BMI	< 25	108	NA	NA	0.907	NA	NA	0.491
	≥ 25	20	NA	NA		NA	NA	
Neoadjuvant chemotherapy	yes	41	849	359–1339	** < 0.001**^a^	875	616–1134	** < 0.001** ^a^
	no	87	NA	NA		NA	NA	
Adjuvant chemotherapy	yes	11	1120	716–1524	** < 0.001** ^a^	1285	784–1786	** < 0.001** ^a^
	no	117	NA	NA		NA	NA	
Stage (UICC 7th)	I	62	NA	NA	**0.001** ^a^	NA	NA	** < 0.001** ^a^
	II/III/IV	66	1430	264–2596		1811	NA	
Histlogical type	poor/sig	64	NA	NA	0.199	NA	NA	0.467
	well/mod/other	64	2291	NA		NA	NA	
Vascular invasion	negative	47	NA	NA	**0.030** ^a^	NA	NA	**0.005** ^a^
	positive	81	2345	NA		NA	NA	
Lymphatic invasion	negative	41	NA	NA	**0.027** ^a^	NA	NA	**0.005** ^a^
	positive	87	2473	NA		NA	NA	
pT	I/II	72	NA	NA	**0.011** ^a^	NA	NA	** < 0.001** ^a^
	III/IV	56	1430	682–2178		1574	NA	
pN	0	73	NA	NA	** < 0.001** ^a^	NA	NA	** < 0.001** ^a^
	1	55	1187	711–1663		1293	512–2074	
CEA (ng/mL)	< 5	113	NA	NA	0.664	NA	NA	0.641
	≥ 5	15	NA	NA		NA	NA	
CA19-9 (U/mL)	< 35	98	NA	NA	0.895	NA	NA	0.440
	≥ 35	30	NA	NA		NA	NA	
Liver metastasis	presence	15	664	394–934	** < 0.001** ^a^	664	394–934	** < 0.001** ^a^
	absence	113	NA	NA		NA	NA	
Peritoneal dissemination	presence	20	699	305–1093	** < 0.001** ^a^	699	305–1093	** < 0.001** ^a^
	absence	108	NA	NA		NA	NA	
AMIGO2	low	99	NA	NA	**0.004** ^a^	NA	NA	** < 0.001** ^a^
	high	29	977	725–1229		1103	0–2587	

*OS* overall survival, *DSS* disease-specific survival, *CI* confidence interval, *MST* median survival time, *BMI* body mass index, *sig/poor/mod/well* signet-ring cell/poorly/moderately/well differentiated, *pT/N* pathological T/N stage, *CEA* carcinoembryonic antigen, *CA19-9* carbohydrate antigen19-9, *NA* not applicable, ^a^statistically significant

Next, we examined which factors were independent risk factors for OS. The Cox proportional hazards model showed that high AMIGO2 expression was significantly associated with OS (hazard ratio [HR]: 2.383; 95% confidence interval [CI]: 1.316–4.314; *p* = 0.004). The presence of lymph node metastasis (HR: 3.554; 95% CI: 1.749–7.223; *p* < 0.001) was also independently correlated with OS. Conversely, vascular invasion, lymphatic invasion, and pT were not independent prognostic factors for OS (Table 3).

**Table 3:** Multivariate analyses of risk factors for overall survival in gastric cancer patients

parameter	HR	95% CI	*P* value
Vascular invasion	1.134	0.424–3.033	0.803
Lymphatic invasion	0.810	0.271–2.422	0.707
pT	1.123	0.557–2.267	0.746
pN	3.554	1.749–7.223	** < 0.001**^a^
AMIGO2 high	2.383	1.316–4.314	**0.004** ^a^

*GC* gastric cancer, *HR* hazard ratio, *CI* confidence interval, *pN* pathological N stage, *pT* pathological T stage, ^a^statistically significant

### AMIGO2 expression was associated with liver metastasis in GC patients

**Fig. 3:**
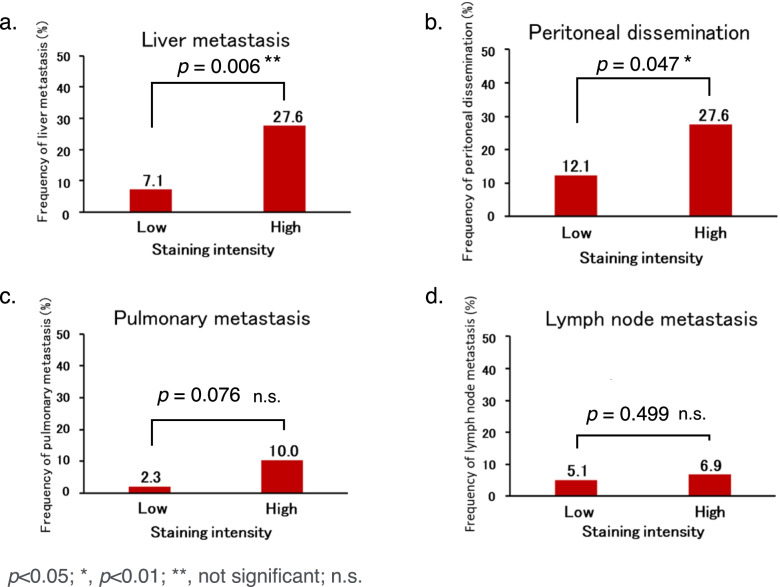
AMIGO2 expression in primary gastric cancer cells and cells from metastatic sites. Increased AMIGO2 expression was significantly associated with increased frequency of (**a**) liver metastasis and (**b**) peritoneal dissemination, but not (**c**) pulmonary metastasis or (**d**) lymph node metastasis. The chi-square test was used for all statistical analyses

Finally, we focused on the potential of AMIGO2 to suggest metastasis in GC patients. To this end, we examined whether AMIGO2 expression had any relationships to sites of metastasis; thus, incidences of liver metastasis and peritoneal dissemination were evaluated in the cohort. Then, the relationship between AMIGO2 expression in GC tissues and distant metastasis was compared by the chi-squared test. The frequency of liver metastasis (*p* = 0.006, Fig. 3a) and peritoneal dissemination (*p* = 0.047, Fig. 3b) was higher in the high AMIGO2 expression group than in the low expression group. Meanwhile, there was no significant difference in lung metastasis (*p* = 0.076, Fig. 3c) or lymph node metastasis (*p* = 0.499, Fig. 3d) according to AMIGO2 expression level.

In the multivariate analysis of risk factors for liver metastasis in GC patients, high AMIGO2 expression was significantly associated with liver metastasis by the logistic regression analysis (odds ratio [OR]: 4.308; 95% CI: 1.212–15.314; *p* = 0.024). Conversely, vascular invasion, lymphatic invasion, and pN were not independent prognostic factors (Table 4).

**Table 4:** Multivariate analyses of risk factors for liver metastases in gastric cancer patients

parameter	OR	95% CI	*P* value
Vascular invasion	1.609	0.727–3.565	0.241
Lymphatic invasion	1.198	0.489–2.938	0.693
pN	6.065	0.793–46.401	0.083
AMIGO2 high	4.308	1.212–15.314	0.024*

*GC* Gastric cancer, *OR* odds ratio, *CI* confidence interval

In the multivariate analysis of risk factors for peritoneal dissemination in GC patients, the logistic regression analysis showed no significant difference between high AMIGO2 expression and the frequency of peritoneal dissemination (OR: 3.082; 95% CI: 0.942–10.085; *p* = 0.063). There was also no significant difference in the frequency of peritoneal dissemination among patients divided by vascular invasion, lymphatic invasion, pT, or pN (Table 5).

**Table 5:** Multivariate analyses of risk factors for peritoneal dissemination in gastric cancer patients

parameter	OR	95% CI	*P* value
Vascular invasion	0.723	0.343–1.524	0.394
Lymphatic invasion	1.839	0.870–3.887	0.111
pT	2.110	0.551–8.082	0.276
pN	2.835	0.610–13.186	0.184
AMIGO2 high	3.082	0.942–10.085	0.063

*GC* gastric cancer, *OR* odds ratio, *CI* confidence interval, *pN* pathological N stage, *pT* pathological T stage

## Discussion

The pathogenesis of metastasis involves many steps and depends on the intrinsic nature of the cancer cells and host responses. In 1889, the association between host and cancer cells was first revealed by Paget [20]. The “seed and soil” hypothesis is still widely accepted, wherein, the “seed” is recognized as a progenitor cell, initiating cell, cancer stem cell, or metastatic cell, and the “soil” is defined as a host factor, stroma, niche, or tissue microenvironment [21].

In terms of modern molecular oncology, it has been well established that epithelial-mesenchymal transition [22], receptors such human epidermal growth factor receptor 2 (HER2) [23], and microRNAs [24] regulate tumour metastasis. Kanda et al. demonstrated that AMIGO2, a member of the AMIGO family, functions as a driver gene for liver metastasis in a mouse model of fibrosarcoma [25]. They found that knocking down AMIGO2 expression tumour cells obtained from mice with high liver metastatic potential weakened the adhesion of tumour cells to hepatic vascular endothelial cells and suppressed liver metastasis. Conversely, forced expression of AMIGO2 in non-hepatic metastatic tumour cells enhanced their adhesion to hepatic vascular endothelial cells, resulting in the formation of liver metastases. Meanwhile, there was no increase in the adhesion of tumour cells to lung endothelial cells or in the formation of lung metastases. Thus, they showed that AMIGO2 drives liver metastasis by homophilic/heterophilic adhesion of tumour cells to liver endothelial cells [25]. Accordingly, our previous study showed that in human colorectal cancer, AMIGO2 expression was correlated with liver, but not lung or peritoneal, metastasis [15, 18]. In our data, AMIGO2 expression was significantly higher in patients with vascular invasion (Table 1). It suggests that AMIGO2 expression may imply the vascular affinity. In general, GC forms metastases via three distinct mechanisms including: i) lymphatic, ii) hematogenous, and iii) dissemination. Each of these metastatic mechanisms can be enhanced by numerous molecular processes such epithelial-mesenchymal transition, genetic mutations, and epigenetic alterations.

In this study, we performed immunostaining for AMIGO2 in specimens from GC patients to investigate its impact on prognosis and various metastatic outcomes. To date, there have been only four reports of AMIGO2 immunostaining in tumour tissues, human melanoma [26] and our previous studies on colorectal and gastric cancers [15, 18, 25]. Gastrointestinal tumours tend to form liver metastases more than other malignancies, especially colon cancer, which tends to metastasize to the liver more frequently than rectal cancer via portal vein reflux. Conversely, the frequency of liver metastasis is lower in GC than in colon cancer, despite portal vein reflux. Nevertheless, it is important to understand the underlying mechanisms of liver metastasis in GC and how to treat liver metastases because of the worse prognosis of GC patients with liver metastases.

In this study, we determined that AMIGO2 expression in GC tissue was an independent prognostic factor for OS. We also found that high AMIGO2 expression was significantly related to liver metastasis in GC patients. This result is consistent with previous observations that AMIGO2 increased cancer cell adhesion to the hepatic vascular endothelium [15] and of increased hepatic metastasis in GC patients with high AMIGO2 expression [15].

Regarding peritoneal dissemination, there was a significant difference in the univariate analysis but not in the multivariate analysis, so it should be noted that it was not an independent prognostic factor in this study. However, other factors such as cadherin may be intricately related to peritoneal dissemination, and it remains possible that AMIGO2 could be involved in peritoneal dissemination. Several adhesion molecules such as E-cadherin, integrin, and dihydropyrimidinase-like 3 (DPYSL3) have been found to be involved in peritoneal dissemination [27]. Therefore, although a relationship between AMIGO2 and peritoneal dissemination has not yet been clarified, it remains a possibility.

Univariate analysis revealed that AMIGO2 expression was significantly correlated with both OS and DSS in GC patients. Taken together, it may be possible that AMIGO2 expression worsens the prognosis of GC patients by increasing their likelihood of suffering liver metastasis.

Recently body fluids including liquid biopsy, circulating tumour cells, cell-free nucleic acid, circulating tumour DNA, and carcinoembryonic antigen have been investigated for developing new biomarkers in GC [28–30]. However, immunohistological analysis remains essential for analysing cancer progression, invasion, and various tumour properties. In this study, we found that AMIGO2 expression in primary GC tumours correlated with the frequency of liver metastasis.

## Conclusions

In conclusion, this study showed that AMIGO2 is induced in GC tissues and can mediate liver metastasis. We also found, for the first time, that immunohistochemical detection of high expression of AMIGO2 can predict the prognosis of GC patients. Determining AMIGO2 expression in GC can help predict patient prognosis and the incidence of liver metastasis, and AMIGO2 may provide a novel biomarker to GC.

## Supplementary Information

**Additional file 1.**

## Data Availability

The datasets used and/or analysed during the current study are available from the corresponding author on reasonable request.

## Abbreviations

GC: Gastric cancer
OS: Overall survival
DSS: Disease-specific survival
HR: Hazard ratio
CI: Confidence interval
OR: Odds ratio
HER2: Human epidermal growth factor receptor 2
DPYSL3: Dihydropyrimidinase-like 3
