# Supplementary material for: The impact of AMIGO2 on prognosis and hepatic metastasis in gastric cancer patients
Source: BMC Cancer. 2022 Mar 16;22:280. doi: 10.1186/s12885-022-09339-0 (PMC8925171; doi:10.1186/s12885-022-09339-0)
Supplement: Supplementary file 1 — Additional file 1. [file 12885_2022_9339_MOESM1_ESM.pptx]

## Slide 1
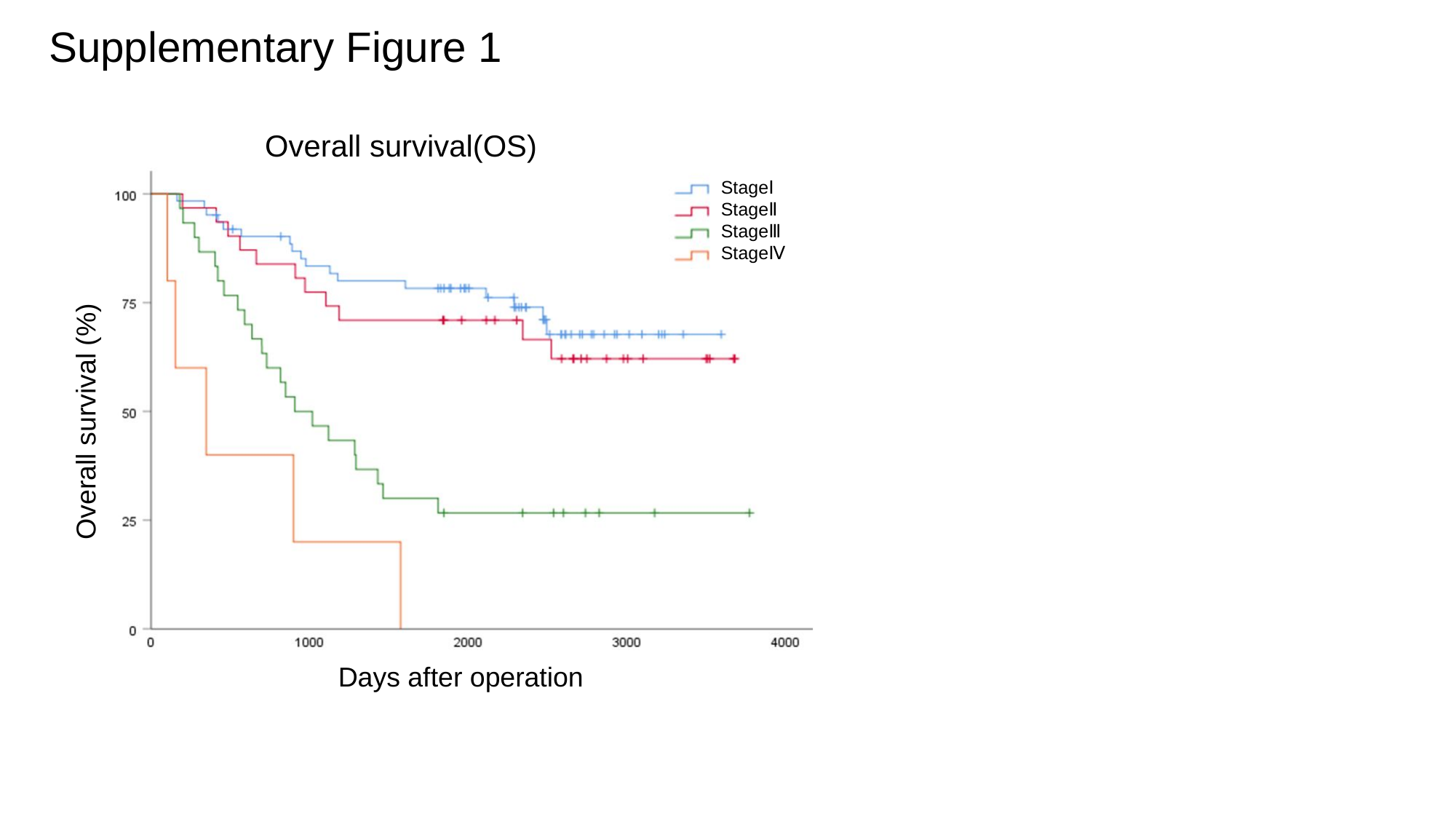

Supplementary Figure 1
Overall survival(OS)
Overall survival (%)
Days after operation
StageⅠ
StageⅡ
StageⅢ
StageⅣ
Log-Rank
p = 0.004
